# A Qualitative Evaluation of Hand Drying Practices among Kenyans

**DOI:** 10.1371/journal.pone.0074370

**Published:** 2013-09-19

**Authors:** Bobbie Person, Katharine Schilling, Mercy Owuor, Lorraine Ogange, Rob Quick

**Affiliations:** 1 Office of the Director, National Center for Emerging and Zoonotic Infectious Diseases, Centers for Disease Control and Prevention, Atlanta, Georgia, United States of America; 2 Division of Foodborne, Waterborne, and Environmental Diseases, National Center for Emerging and Zoonotic Infectious Diseases, Centers for Disease Control and Prevention, Atlanta, Georgia, United States of America; 3 Kenya Medical Research Institute, Kisumu, Kenya; University of Pittsburgh Medical Center, United States of America

## Abstract

**Background:**

Recommended disease prevention behaviors of hand washing, hygienic hand drying, and covering one’s mouth and nose in a hygienic manner when coughing and sneezing appear to be simple behaviors but continue to be a challenge to successfully promote and sustain worldwide. We conducted a qualitative inquiry to better understand current hand drying behaviors associated with activities of daily living, and mouth and nose covering practices, among Kenyans.

**Methods and Findings:**

We conducted 7 focus group discussions; 30 in-depth interviews; 10 structured household observations; and 75 structured observations in public venues in the urban area of Kisumu; rural communities surrounding Kisumu; and a peri-urban area outside Nairobi, Kenya. Using a grounded theory approach, we transcribed and coded the narrative data followed by thematic analysis of the emergent themes. Hand drying, specifically on a clean towel, was not a common practice among our participants. Most women dried their hands on their waist cloth, called a leso, or their clothes whether they were cooking, eating or cleaning the nose of a young child. If men dried their hands, they used their trousers or a handkerchief. Children rarely dried their hands; they usually just wiped them on their clothes, shook them, or left them wet as they continued with their activities. Many people sneezed into their hands and wiped them on their clothes. Men and women used a handkerchief fairly often when they had a runny nose, cold, or the flu. Most people coughed into the air or their hand.

**Conclusions:**

Drying hands on dirty clothes, rags and lesos can compromise the benefits of handwashing. Coughing and sneezing in to an open hand can contribute to spread of disease as well. Understanding these practices can inform health promotion activities and campaigns for the prevention and control of diarrheal disease and influenza.

## Introduction

Preventable diarrheal disease contributes to an estimated 4.2% of the total DALY global burden of disease in low and middle income countries and is responsible for the deaths of an estimated 801,000 children under 5 years of age every year [1], [2]. In Kenya, approximately 9% of all under-five child deaths are attributable to diarrheal disease [3]. Proper hand hygiene is one of the most effective measures in preventing and controlling the spread of disease [4]. In a recent meta-analysis, hand hygiene was found to reduce diarrheal disease by 31% and respiratory disease by 21% [5]. The recommended way to wash hands includes a step-wise process that involves wetting the hands with water, applying an ample amount of soap, thoroughly rubbing all surfaces of the hands together with soap and water for at least 20 seconds, rinsing the hands with water, and finally drying the hands using a clean towel or letting the hands air dry [4].

Hand drying is an important step in the handwashing process that is often under emphasized [6]. The action of drying hands is important as wet hands more easily transmit microbial contaminants as compared to dry hands [7], [8]. Additionally, the action of rubbing hands on a clean towel during the drying process creates friction that allows for the removal of microbial contaminants [8].

Research comparing the effectiveness of various hand drying methods is inconsistent and is especially sparse in resource limited settings where common hand drying methods found in industrialized countries, such as the use of a clean cloth, disposable paper towels, or warm air driers, are often not available. The hand drying options for people living in these settings might more realistically include air drying or using available cloth or clothing. The published literature often does not include air drying (evaporation) as a studied method of hand drying. To our knowledge only one evaluation exists which included air drying as a possible hand drying method. This evaluation, which took place amongst volunteer participants willing to have hands artificially inoculated with *Micrococcus luteus*, compared the effectiveness of four hand drying methods (air drying or evaporation, warm air dryers, cloth towels, or paper towels); evaluation results revealed no significant difference between the number of *M. luteus* colony forming units on hands and the method of hand drying used [9].

In a community-based observational handwashing survey of households living in two urban settlements in India, it was found that before the implementation of a handwashing intervention, 42% of respondents from one settlement and 37% from another reported drying their hands using clean materials. After the handwashing intervention, researchers found that the use of clean material to dry hands significantly increased in both settlements to 67.9% and 93%, respectively. The researchers suggested that future handwashing programs should encourage, among other things, hand drying with a clean material [10]. However, this evaluation did not identify the type of clean material that was used by respondents to dry their hands. Because evidence on hand drying practices in resource limited settings is scarce, we chose to explore the topic further to characterize the risk behaviors associated with current hand drying practices, identify the barriers to optimal hand drying, and make recommendations to improve hand drying practices and reduce disease.

## Methods

### Setting

This qualitative inquiry was conducted predominately in rural Nyando District, Nyanza Province Kenya. In Nyando District (population 400,000) the majority of the population is Luo and earns its income through subsistence farming, cultivating maize, sorghum, cassava, and millet; carrying out animal husbandry; and engaging in migrant labor [11]. Many families engage in polygamy and live in “dalas” that consist of a single main house surrounded by 1 to 3 additional households [11]. These dalas are often multigenerational. Data were also collected in the urban slum, Kibera, located 7 km southwest of Nairobi, with an estimated population of 170,000 [12]. People in Kibera live in sub-optimal housing with insufficient access to clean water and sanitation [13], [14]. These resource-poor communities were chosen due to their high risk for diarrheal disease and influenza.

### Design and Recruitment of Participants

We conducted 7 focus group discussions (FGD) with a total of 45 participants; 30 in-depth interviews; 10 structured household observations; and 75 structured observations in public setting such as markets, restaurants, minibuses, and on the street among Kenyans living in Nyando District of Nyanza Province and Kibera slum in Nairobi area. Due to the paucity of information on hand drying practices, we chose a qualitative approach to better understand specifically hand drying practices associated with various activities of daily living along with barriers to optimal hand drying practices, with recommendations for improving such practices and reducing disease threats. We used a modified grounded theory approach with an emergent qualitative thematic analysis allowing the hypothesis to be generated from the data [15], [16], [17]. The coding structure evolved inductively with the narrative data of earlier interviews informing subsequent interviews over time supplemented with field notes [15], [16], [17].

We used purposive sampling to recruit people, especially reproductive age women with young children, who would be engaging in typical activities of daily living in rural, peri-urban, and urban settings, for in-depth interviews, household observations, and FGD [18]. Women were recruited through a local community-based organization with ties to the community. We focused attention on resource-poor areas which would benefit significantly from optimal handwashing and drying practices to prevent and reduce infectious disease. Systematic observation and recording procedures along with a proportional sampling framework were developed to identify women and men for structured public participant observations in such settings as markets, public transportation, and restaurants. Procedures for the structured observations identified who was to be observed, when and where they were observed, what was to be observed, and how the observations were to be recorded [19]. Illustrative scenarios were also captured in field notes.

### Data Collection

Data collection was conducted from July to September 2010. We conducted 5 rural and 2 peri-urban based focus group discussions (FGD); 30 in-depth interviews (IDI) with women and men; 10 IDI of women in households which included structured household observations (SHO); and 75 structured observations in public venues (SPO). The field team consisted of a senior behavioral scientist and three Dhuluo and English speaking, Kenyan research assistants who had previous experience with qualitative methods within the communities where the assessment was conducted. University trained qualitative research assistants (co-authors MO & LO), well known to the community, served as the primary data collectors and logistic coordinators setting up focus groups and interviews within the communities. Households in Kisumu were identified with the assistance of Safe Water and AIDS Project (SWAP) staff along with field officers known to SWAP staff in Kibera. SWAP is a non-governmental organization based in Western Kenya that engages HIV support and self-help groups to promote and sell water treatment and other health products as an income generating activity that also benefits the wider community [11]. Women who were members of SWAP groups were listed and then randomly chosen to be recruited. If they agreed to participate, they were given the place, day and time of the interview or group discussion. Following the interview, participants received a small thank you gift for their time and participation. We had no refusals.

FGD and IDI topic guides were developed, pretested, and modified to adapt to local linguistic and cultural nuances. Topic guides included hand washing and drying behaviors associated with cooking, eating, diaper changing, and caring for an ill person. Additionally, we explored mouth and nose covering practices associated with coughing, sneezing, and nose blowing. The interview and FGD typically started out with the question, *“Please describe for me all the times during the day that you wash and dry your hands.”* Additional probes allowed for deeper exploration of the topics that emerged supporting areas of interest. Research assistants conducted the interviews and FGD in Dholuo, the local language. Interviews, which were 45–60 minutes in length, were typically conducted in the person’s home and usually only with the interviewer and note taker present. Verbatim field notes were handwritten during the interviews and reviewed during debriefing sessions to verify accuracy of the interview session. Narrative data were transcribed into English with review following translation to ensure accurate translation and local meanings. Transcripts were entered into Atlas-ti, © (ATLAS.ti Scientific Software Development GmbH, Berlin, Germany) as a Word © (Microsoft Corporation, Redmond, WA, USA) document for data management and analysis.

A systematic structured observation guide was also developed, pretested, and modified to capture critical observations of the same behaviors in households and public venues. Staff conducted practice observation sessions in local venues to pretest, clarify, and revise the observation guide and test validity of the guide. Analysis was conducted in the same fashion as described above.

### Data Analysis

English transcripts were entered as Microsoft Word© documents into Atlas-ti© to facilitate text searching, data coding and analysis. Due to the paucity of research on hand drying we used modified grounded theory [15], [16], [17]. Data analysis began with the first interview and FGD allowing for emerging, unexpected, and/or inconsistent issues to be explored in subsequent interviews and FGDs. Due to time constraints and ongoing data collection tasks the primary author (a behavioral scientist with experience in qualitative research) was the primary data coder with verification of interpretive codes by the research assistants. She used open, axial, and selective coding to analyze the FGD and IDI narratives [18]. A coding frame was developed through open coding, a word-by-word analysis, used to identify, name, and categorize explanations and descriptions of the day-to-day reality of participants as related to hand washing and drying as well as towel or handkerchief use. Consensus on the coding frame was obtained through discussions with the qualitative research assistants, who were from the local communities and conducted the original interviews. Axial coding, the process of relating codes to each other, via a combination of inductive and deductive thinking, was used for analysis of specific emergent themes, across themes, and for the relationships between themes. Over the course of data collection, emergent themes became redundant, suggesting that all major themes had been identified. An analysis matrix served as a framework for the resulting findings.

The trustworthiness of our data was derived from standardization of methods and documentation for auditability, triangulation of the data, and verification of data findings with local staff members who live amongst those we interviewed. A standardized implementation document guided the qualitative methodology with all procedures, topic guides, informed consents, timelines, interview schedules, data collection strategies, data management, and analysis strategies written out. Process data was collected to allow for auditability of the process. Triangulation of data was derived through the multiple data collection methods (interviews, focus group discussions, and structured observations); multiple perspectives (women and men); multiples venues (private home-based and public venues); and a systematic literature review on hand drying practices in resource poor communities. Findings were verified amongst local SWAP staff that live within our study communities as well as corroborating results with similar findings across settings. There is a potential for bias by having only one coder, which we attempted to manage by discussing findings along each step of the process with local team members.

### Ethics Statement

The Centers for Disease Control and Prevention Human Research Protection Office (HRPO) and institution review board (IRB) determined that these project activities are exempt under 45 CFR 46.101(b)(2) and issued a written waiver. Local Ministry of Health and political authorities provided permission to carry out the project. HRPO and IRB approved the informed consent process conducted with all participants who took part in FGD, interviews, and household observations. Due to limited ability of participants to read and write the informed consent was available in both English and Dhlou to be read aloud by bilingual research staff and participants provided a verbal consent, with the consent acknowledged with the signature on the informed consent document of a witness present at the time. Research staff reviewed the consent process and all consent forms to ensure compliance with the process. Structured public observations were not consented because no contact was made with individuals and no personal identifiers were collected.

## Results

### Characteristics of Participants

Participants included mothers with children under five, housewives, teachers, clinic staff, men and other household members, office workers, petty traders and sellers, farmers, and food handlers. Overall, there were a greater number of women in the inquiry because they perform most of the duties in the home. FGD participants (N = 45) were 99% female, ranging in age from 17–40 years old; 84% were married with an average of 1 (0–3) child under 5 years old. Participants in in-depth interviews (N = 30) were 67% female, ranging in age from 19–43 years, with an average of 2 children (0–4) under 5 years old. Similarly, women who we observed in their homes (N = 10) ranged in age from 19–40 years old, were all married, and had on average 2 (1–4) children under 5 years old. Additionally, we conducted observations of people (N = 75) in public venues; 67% were women.

### Emergent Themes of Interviews and Focus Groups: Household Behaviors and Practices

Overall, most women either don’t dry their hands or, if they do, generally dry them on their “leso” [an inexpensive cloth they wrap around their waist like a skirt or apron] or their clothes when cooking and working in the kitchen. A woman said, “*Whenever I have a leso cloth tied around my waist I normally use it to wipe my hands. It means that when I am in the kitchen and I want to cook I wash my hands and then use the leso cloth to wipe my hands.” (FGD1: R5).* Some women use towels or rags that are specifically used in the kitchen for wiping hands. A few women described wiping their hands on the curtain in their kitchen which often serves as a wall between sleeping areas and the kitchen area. Another woman said, “*After washing my hands when I want to cook, I dry them using any cloth I have near me like my dress, a rag, even a curtain. I use anything nearby.” (FGD3: R3).* Some women use a “kitamba,” an all-purpose cloth or purchased handkerchief which is often carried in the waist of the leso, to wipe the nose or to dry hands when working in the kitchen.

The typical kitchen was often in a confined space lacking hand washing facilities and other amenities. Water is carried into the home and stored in large containers. Cooking typically took place on a three-stone open fire pit. Women often rinsed their lesos, rags, or towels in basins or in the river, a nearby water source. All women told us that they would prefer to have a kitchen towel and would use it if it was affordable. Women described the need for a loop on a large towel so that it could be hung in a central location in the kitchen for ease of use.

### Emergent Themes of Interviews and Focus Groups: Before and After Eating

People reported two different sets of behaviors associated with hand washing and drying when eating. A typical meal is eaten with ones’ fingers so people wash their hands prior to eating but rarely dry their hands on a towel or cloth. They usually air dry them or begin eating with wet hands unless there is a visitor at the table. If a visitor is at the table a few women described having a small towel or rag for hand drying prior to eating: “*If I have a visitor, I give him or her towel to wipe the hands with. On that day everyone at the table will use a towel. After we are done eating I also pour water for people to wash their hands and then they use the same towel to wipe their hand. We only use a towel when there is a visitor.”(FGD4: R7)* Some people who described washing their hands after eating reported they would air dry their hands, or wipe their hands on their trousers or dress; few reported using a towel. Many people reported that hand washing after a meal was contingent upon the type of food eaten. If the food was smelly or greasy the hands would be washed but, if not, respondents often reported that they would just rub their hands together or wipe them on their clothes. A woman reported, *“You find that most of the time people wash hands after eating depending on the type of food they have eaten. If it is food that is not sticky on hands, you just rub your hands together and that is it. If the food has smell like fish you wash with soap and water and air dry or sometimes rub them on your clothes.” (FGD5:R7)*. A few people expressed concern that the towel they might use to dry their hands would be carrying germs and described air drying their hands as the preferred safe behavior.

### Emergent Themes of Interviews and Focus Groups: Latrine Use

People reported infrequent hand washing and hand drying behaviors following latrine use. One woman reported, *“After visiting the latrine I do nothing to my hands. I don’t wash my hands. I do not want to lie. I am just telling you what I am doing because even getting that water is not easy. Like during the drought we have to go far to get the water from a certain borehole since the ponds get dried up.” (R34)* The typical latrine is often located away from the house without water for hand washing nearby. Men specifically made a distinction between “short call” [urination] and “long call” [bowel movement] reporting that it was not necessary or convenient to wash one’s hands after short call and that, even though they knew to wash after long call, they rarely, if ever, did. They reported that the distance between hand washing facilities and the latrine, the lack of water and hand washing supplies, and inconvenience were the main barriers to hand washing and drying after latrine use. A man reported, *“I do not wash my hands nor dry them after visiting a latrine. In most cases the latrines that I visit do not have water for washing hands but even if I am at home I do not wash my hands after visiting the latrine. There is no reason at all for not washing. If I go for a short call my hands will just be clean and if I go for a long call then I use the leaves of a tree or even an old newspaper then the hands will not be dirty because I will not have touched the feces with my hands. So for me to be sincere I do not wash my hands after visiting the latrine.” (R29)* People did report that they would be more likely to wipe their hands after latrine use if there was a towel hung on the latrine just for that purpose.

### Emergent Themes of Interviews and Focus Groups: Diaper Changing and Cleaning a Child

While many women reported washing their hands after changing a dirty diaper, few actually dried them. Those women who reported drying their hands dried them on their leso or their clothes: *“After washing the dirty nappies I wash my hands with soap but it is not a must for me to dry them. If I am going to touch something and I am in a hurry then I just wipe on my skirt.” (R27).* Women who did not dry their hands reported air drying their hands or doing nothing. Several women reported that they cleaned their hands in the same water they washed the diaper. A woman reported, “*What I normally do is to prepare water in a basin and soap also. I then wash the baby’s butts and pour that water in the latrine. I then come back and wash the nappy that was soiled and then in the process wash my hands before I go and put some clothes on for the baby. When the baby poops a lot I end up washing the whole body but if it is just a little then I just wash the butt alone. (FGD1:R4)* A few women reported that circumstances and inconvenience contributed to their not washing their hands. Women reported that if they were in a clinic, on mini-bus, or in the garden working they did not wash their hands because there was no soap and water, they just wiped their hands on their leso or their clothes. A woman told us, *“If he has passed stool and I am in the clinic I will not wash my hands, where will I get water? I will just wipe with the nappy and forget about washing hands. (R9)* Most reported knowing that they should wash their hands.

### Emergent Themes of Interviews and Focus Groups: Caregiver Behaviors

Most women reported that they wiped their hands on their leso or their dress or air dried their hands when caring for a sick person. Some women reported washing their hands but then they dried them on their leso: “*I make sure I handle the sick person hygienically by washing my hands before and after handling her. I dry my hands on my leso. (FGD 2:R6)* Even men who cared for their wives typically used the woman’s leso to dry their hands. One man told us, “*I cared for my wife once. After cleaning her there was a leso cloth that she had carried with her and I would use it to wipe my hands after washing them. I would then hang it outside to dry. This particular leso was never washed until she got discharged and then it was washed with the clothes that she had in the hospital.” (R33)* No one mentioned using anything else when caring for a sick person.

### Emergent Themes of Interviews and Focus Groups: Coughing, Sneezing and Nose Blowing

People described distinctions between blowing their nose after spontaneous sneezing and when they had cold symptoms with a runny nose or persistent mucus. Sneezing was characterized as a spontaneous action where people rarely used a handkerchief. People typically reported sneezing into their hands and wiping their hands on their clothes or rubbing their hands together until they were dry. Some reported turning the head away from others and blowing their nose openly into the air. One person said, “*Most of us will just move away and blow your nose in the air then wipe your hands on your clothes (laughter).” (FGD5:R8).*

Most people reported that they did blow their nose on a rag, handkerchief, or other type of cotton fabric when they had cold or flu symptoms, or when producing mucus. Some men and many women carried a handkerchief with them when traveling to town, church or other social functions. Men reported preferences of handkerchiefs of light cotton that were absorbent, soft, and fold nicely to carry in their pants pocket. Women also preferred handkerchiefs but did report using old rags, cut up T-shirt material, and their leso to blow their nose on. Women often purchased used handkerchiefs and towels from a vender in the market. Many women reported blowing their children’s noses on the child’s clothes or their own leso. Many women also pinned rags or cut up cloth to the shirt of young children when they had a runny nose so the “handkerchief” would not get lost. Children were often portrayed as having excessive mucus and in need of a more absorbent handkerchief. A woman described caring for her children with a cold, “*When I need to blow my nose I use a handkerchief to blow my nose. At times I also use the leso cloth. As for the children, I normally cut rags for them from an already worn out cloth. I normally tend to look for cloths that are of cotton material. When they really have a bad flu, I use a safety pin to hold the handkerchief on their chest such that if they are playing outside, then they can wipe their nose. And when I am near them, I can also wipe for them and not use their tops that would look dirty if I used it to wipe their nose. Would you want to be close to a child with a very dirty top, full of mucus? (laugh) I don’t think so.”(R9)* Women reported the need to regularly wash the handkerchiefs.

### Household Observations

We conducted structured observations for hand washing and drying behaviors associated with normal activities of daily living among 6 rural and 4 urban women in their homes. Eight of the 10 women had co-wives in their households. Three women had tap water and 7 gathered their water from a well, pond, or river. Eight women had soap and 4 had towels. We observed 7 women during food preparation and cooking; 5 following use of latrine; 7 changing a dirty diaper; 7 before and after eating; and 5 washing dishes. The household observations allowed us to triangulate the data, confirming our FGD and IDI findings. A scenario is provided below for the reader to get a sense of an observation:

*The woman had a 10–month-old baby girl who had defecated. She was cleaning the child. She removed the nappy and wiped the baby’s bottom with the same dirty nappy and left her with the child of her co-wife to continue playing on a mat. She did not wash the baby. She then took the nappy to the latrine to throw out the feces. When she came back, she dipped the nappy inside a basin, rinsed her hands in the same basin with the nappy and then wiped them on her leso as she walked over to talk with us. She never washed her hands.*

### Structured Public Venue Observations: Hand Hygiene

We observed 24 adult men and 51 adult women in a public setting. All individuals observed would have benefited from public health hand-hygiene recommendations to wash their hands to prevent the transmission of disease after the activity observed. Of the 75 individuals observed, 51% had access to a nearby water source for handwashing, although the water supply could not be considered safe; 55% to soap located within reach; and 32% to a towel at the time of the observation. We observed 35 people engaging in additional activities of daily living where it would be favorable to have washed their hands, including eating, changing a baby’s diaper, returning from the garden, and occupational activities. Four people (11%) washed their hands with soap and water and dried with a towel; 2 (6%) people washed with soap and water and dried their hand on their clothes or leso; 15 people (43%) who did not wash their hands wiped their hands on their leso or clothes; 3 people (9%) wiped their hands on a handkerchief; and the remaining 11 people (31%) rubbed their hands together or did nothing to clean their hands.

### Structured Public Venue Observations: Coughing and Sneezing

We observed 30 people sneezing or blowing their nose: 2 people (6%) sneezed into a handkerchief; 17 (57%) used a leso or their clothes to either sneeze into or wipe mucus from their nose after a sneeze; and the remaining 11 (37%) sneezed into their hands or the air, wiping their hands on a chair, stair rail or other inanimate object and then returned to the activity they were engaged in before the sneeze. Some of the observations were parents wiping the noses of their young children. We also observed 10 people coughing. Nine people (90%) coughed into their hands or the air; one person coughed into a handkerchief. Of the 9 who coughed into their hands 4 (40%) people wiped their hands afterward on their leso or clothes.

## Discussion

Results of this in-depth, qualitative investigation of hand drying practices, suggested that, despite several global initiatives to promote handwashing through multiple venues [20], hand drying practices in this population were sub-optimal and appeared to defeat the purpose of handwashing by exposing hands to potentially contaminated objects. Few people were observed to air-dry their hands, which, in fecally contaminated environments, is the most consistent recommendation if a clean towel is not available [4], [20]. There appeared to be little awareness of specific hygiene standards associated with hand drying behavior, which was typically determined by convenience. For example, most women dried their hands on their leso or their clothes when cooking, eating, or after rinsing hands in water used to rinse diapers, with little apparent concern about the level of cleanliness of the fabric. Only one woman in our inquiry used a clean towel. This was the cook for an outdoor eating establishment in which the towel could have been provided for her to use. Men typically dried their hands on their trousers or a handkerchief. Children rarely dried their hands after washing, but, if they did, usually wiped them on their clothes.

Our findings identified several barriers to optimal hand drying practices. First, we observed that, while air drying is free and universally “accessible”, it is inconvenient, takes time, and wet hands interfere with daily activities. Second, the availability of clothing, a convenient, inexpensive option for immediate hand drying, may have reduced the motivation to develop other approaches. Third, handwashing facilities were not conveniently located and, when present, often lacked the necessary supplies (water, soap, and clean towel), prompting people to wipe their hands on clothes, dirty rags, or rub them together to dry. This barrier was most pronounced near toilet facilities, where concern about theft of soap or towel was a major disincentive to the installation of hand washing stations. Consequently, most subjects did not wash their hands after using the latrine, preferring to just wipe them on their clothes or nearby objects.

Managing coughing and sneezing was a special case. Although it is recommended to cough or sneeze into the crook of the elbow or a handkerchief [21], this message had apparently not reached this population. Instead, subjects most frequently reported coughing or sneezing into the open air, their hand, leso, or a handkerchief, and none of them reported washing their hands after possible exposure to a cough or sneeze. Clearly there is need for an for improved health intervention to specifically address hand drying practices associated with contaminated clothes to decrease risk of upper respiratory illnesses and influenza.

There were several limitations to this inquiry. While not a limitation, because we used a purposive, convenience sample in just two regions of Kenya, one must remember that this population was not necessarily representative of the communities in which the inquiry took place, and the results are not generalizable. The public observations provided triangulation of data to suggest that there were similarities across behaviors of people who were not aware of being observed and persons we observed in their homes. The behaviors we assessed appeared to be nearly universally practiced and the lessons learned could be used to tailor messages for future hygiene programs. That being said, there may have information bias during household interviews and focus group discussions as interview subjects may have provided answers that they believed the interviewer expected to hear. This limitation was mitigated through direct observations in households and in public that revealed prevalent practices. Finally, among persons who were being observed, there may have been a Hawthorne effect, whereby people improved their typical hygiene behaviors while being observed. This effect was mitigated by direct observations in public settings of persons who were not aware of being observed that revealed a consistency of hand drying behaviors across several locations.

This inquiry points to a need for hygiene campaigns to address hand drying specifically to assure that handwashing has the desired impact. Hand hygiene is typically promoted with an emphasis placed on the use of soap and water as well as lathering all surfaces of the hands thoroughly with less emphasis on hand drying as an important step in the process [6]. While hand drying interventions may be challenging because of the time required for air drying and the time and expense needed to provide clean hand towels, the potential for behavioral cuing towards specific activities of daily living could be beneficial. Our data suggest a need to design handwashing interventions which include hand drying on clean material during specific household activities, including cooking, eating, using a latrine, and washing babies and their nappies. Locating handwashing stations with a clean towel in a visible spot near kitchens and latrines could reduce a key barrier to handwashing. Although alcohol-based hand cleansers and paper towels are potential alternative interventions, cost and logistics likely limit their applicability.

## Conclusions

This qualitative inquiry found that it is common for people to wipe their hands when wet or dirty on whatever material is convenient, most typically clothing, when engaging in activities of daily living. Drying hands on dirty clothes and lesos can compromise the benefits of handwashing. The lack of specific health education and promotion materials and messages associated with hand drying may contribute to the spread of diseases associated with poor hygiene. The dearth of rigorous studies on household level, hand drying techniques suggest the need for intervention studies on convenient, hygienic hand drying interventions tailored to household activities and hygienic coughing and sneezing practices. A better understanding of these practices can inform future health promotion activities and campaigns for the prevention and control of diarrheal disease and influenza in resource poor communities lacking clean water, adequate sanitation, and handwashing facilities.
